# Competition between sympatric wolf taxa: an example involving African and Ethiopian wolves

**DOI:** 10.1098/rsos.172207

**Published:** 2018-05-02

**Authors:** Tariku Mekonnen Gutema, Anagaw Atickem, Afework Bekele, Claudio Sillero-Zubiri, Mohammed Kasso, Diress Tsegaye, Vivek V. Venkataraman, Peter J. Fashing, Dietmar Zinner, Nils C. Stenseth

**Affiliations:** 1 Centre for Ecological and Evolutionary Synthesis (CEES), Department of Biosciences, University of Oslo, PO Box 1066, Blindern, 0316 Oslo, Norway; 2 Department of Natural Resources Management, Jimma University, PO Box 307, Jimma, Ethiopia; 3 Cognitive Ethology Laboratory, German Primate Center, Leibniz Institute for Primate Research, Kellnerweg 4, 37077 Göttingen, Germany; 4 Department of Zoological Sciences, Addis Ababa University, PO Box 1176, Addis Ababa, Ethiopia; 5 Wildlife Conservation Research Unit, Zoology Department, University of Oxford, Tubney House, Tubney, UK; 6 IUCN SSC Canid Specialist Group, Oxford, UK; 7 Department of Biosciences, University of Oslo, PO Box 1066, Blindern, 0316 Oslo, Norway; 8 Department of Human Evolutionary Biology, Harvard University, 11 Divinity Avenue, Cambridge, MA 02138, USA; 9 Department of Anthropology and Environmental Studies Program, California State University Fullerton, 800 North State College Boulevard, Fullerton, CA 92834, USA

**Keywords:** *Canis lupaster*, *Canis simensis*, interference competition, exploitative competition, carnivore conservation

## Abstract

Carnivore populations are declining globally due to range contraction, persecution and prey depletion. One consequence of these patterns is increased range and niche overlap with other carnivores, and thus an elevated potential for competitive exclusion. Here, we document competition between an endangered canid, the Ethiopian wolf (EW), and the newly discovered African wolf (AW) in central Ethiopia. The diet of the ecological specialist EW was dominated by rodents, whereas the AW consumed a more diverse diet also including insects and non-rodent mammals. EWs used predominantly intact habitat, whereas AWs used mostly areas disturbed by humans and their livestock. We observed 82 encounters between the two species, of which 94% were agonistic. The outcomes of agonistic encounters followed a territory-specific dominance pattern, with EWs dominating in intact habitat and AWs in human-disturbed areas. For AWs, the likelihood of winning encounters also increased with group size. Rodent species consumed by EWs were also available in the human-disturbed areas, suggesting that these areas could be suitable habitat for EWs if AWs were not present. Increasing human encroachment not only affects the prey base of EWs, but also may impact their survival by intensifying competition with sympatric AWs.

## 1. Introduction

Carnivore species have coexisted for millennia in many of Earth's ecosystems through temporal, spatial or dietary niche partitioning [1,2]. Over the past several decades, however, herbivore prey depletion resulting from hunting by humans and habitat destruction disrupted their adaptations for coexistence [3]. As a result, many carnivore species face extinction risk because of elevated interspecific competition in shrinking and degraded habitats [4,5]. This competition can take the form of direct lethal encounters, interference competition at kills, exploitative competition over diminished prey populations, exclusion of one species by another from areas of high prey density and fear-mediated shifts to less optimal habitats [2,5].

Two Canidae species, African wolves (*Canis lupaster*) and endangered Ethiopian wolves (*Canis simensis*), coexist in parts of the Ethiopian Highlands [6]. With fewer than 500 adult individuals left in the wild, the Ethiopian wolf (EW) is the world's rarest canid [7]. The African wolf (AW), which was until recently incorrectly regarded as a golden jackal (*C. aureus*) [8], is distributed in northern and eastern Africa [9]. As an ecological specialist and solitary forager with a small population size [6], the EW may be particularly sensitive to the impacts of interference competition from the AW, especially in light of recent preliminary evidence of partial dietary overlap between the two species [10]. Here, we aim to assess dietary overlap, habitat quality and whether interference competition occurs between EWs and AWs in north central Ethiopia.

Relative body mass, group size and territorial ownership are typically the most important factors in determining the outcome of agonistic encounters between carnivores [4,5]. Based on these considerations, EWs (males: 14.2–19.3 kg, females: 11.2–14.2 kg; [11]) should dominate the smaller AWs (males: 9.0 kg, females: 8.1 kg; [12]) in one-on-one agonistic encounters. By recording behavioural interactions in two ecologically distinct zones (buffer zone and core area), we were able to assess the relative importance of these factors in determining the outcome of interactions. This has crucial consequences for understanding potential conservation threats posed to the endangered EW by the AW if both species are forced to share more of their shrinking and degraded habitats across the Ethiopian Highlands.

## 2. Material and methods

### 2.1. Study site

**Figure 1.**
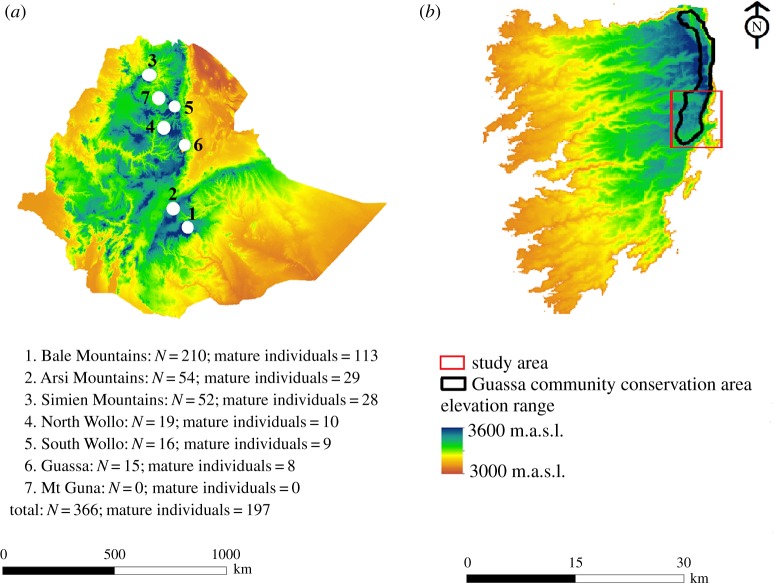
(*a*) Distribution of the seven remaining populations of Ethiopian wolves in the Ethiopian highlands and their respective population sizes (6 = current study area). (*b*) Map of Guassa within the Menz Highlands, north central Ethiopia. The population estimates here are from Marino & Sillero-Zubiri [7].

**Figure 2.**
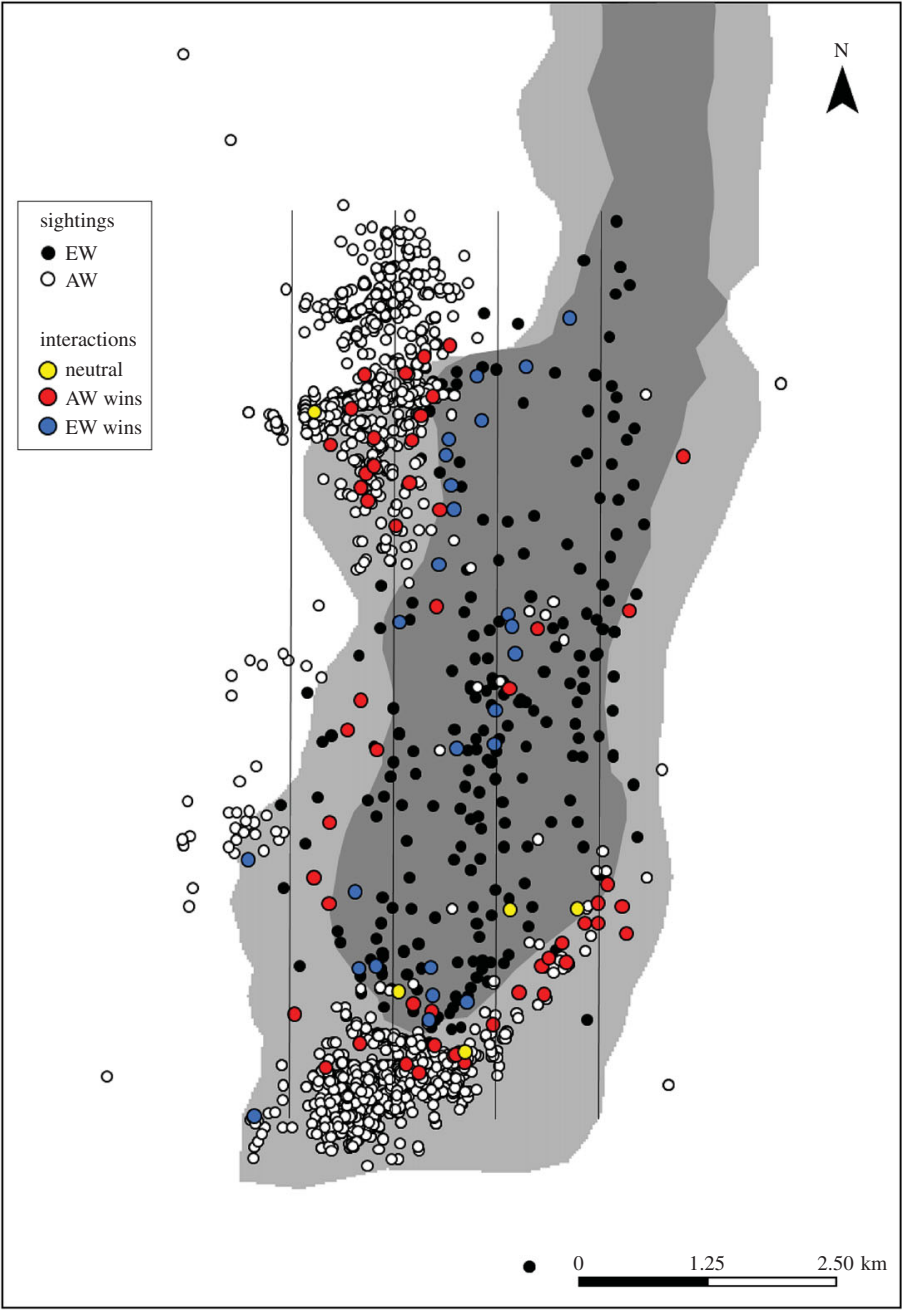
The study area in the southern section of the Guassa Community Conservation Area, including transects (vertical lines) and sighting locations of AWs and EWs. The locations and outcomes of AW–EW encounters are also depicted. The dark grey area indicates the core area, the light grey area indicates the buffer zone and the white area indicates the matrix.

The study was carried out within the Guassa Community Conservation Area (GCCA; figure 1). GCCA contains an unusually intact Afroalpine grassland ecosystem with an estimated 21 EW individuals [13,14]. We delineated the study area into three zones: core area (section of GCCA where all human and livestock activities are prohibited), buffer zone (section of GCCA where controlled livestock grazing is permitted) and *matrix* (human-dominated areas adjacent to GCCA consisting mostly of farmland and settlements) (figure 2). We focused our study on the 30 km^2^ southern portion of the GCCA, within which we regularly spotted eight EWs and 21 AWs.

### 2.2. Observational data collection

Seven AWs from four packs were captured using rubber-padded leg-hold traps and fitted with very high frequency collars (for detailed procedures, see Gutema *et al*. [12]). We used a hand-held directional antenna to locate respective animals, followed them and did focal observations. Locations of the focal wolves were recorded at 30 min intervals during the day time for a total of 3864 h (772.8 ± 323 h) [13]. Data from two collared individuals were excluded from the analysis. One of these individuals was found dead, possibly killed by humans. The other individual was lost during the third month of study when its signal disappeared.

Whenever the two wolf species were observed within approximately 120 m of one another, we recorded the nature of the interaction (neutral, aggression and aggression with bite), the number of individuals of each species present, and the duration and location of the interaction (core area or buffer zone) (cf. [15]). If the interaction was agonistic, we also determined the winner (i.e. which species chased the other away). A *neutral* interaction was recorded if all individuals of both species present ignored one another, an *aggression* was recorded if at least one individual of one species ran towards a member of the other species in an aggressive manner, and an *aggression with bite* was recorded if one or more individuals of one species bit a member of the other species. We used binomial logistic regression to analyse the outcome of winning the agonistic interaction (response variable: which species won in relation to the two sites (categorical explanatory variable: buffer zone and core area)) using the glm function. Given the low population sizes of both EWs and AWs and the fact that both species exhibit territoriality, it is virtually certain that repeated sampling of the same individuals occurred; however, we were unable to account for this in the present study.

### 2.3. Wolf diet

From June to November 2015 and December 2015 to May 2016, EW sightings were recorded while systematically walking four transects (totalling 9 km) thrice monthly. Transects were spaced at intervals of 1 km (figure 2). Since a previous study on the AW diet at Guassa was only based on a three-month study of scats (*n* = 101; [10]), we collected 175 scats during the wet season (June–November) of 2015 and 175 scats during the dry season (December–May) of 2015–2016. The scats were dried and broken into pieces, and prey remains were identified via comparison with reference samples [6]. Data on EW diet, based on frequency of occurrence in faeces, were obtained from a previous 12-month study by Ashenafi *et al*. [13] at Guassa.

### 2.4. Habitat quality estimate

As a proxy for habitat quality, we used rodent density because rodents constitute a major part of the wolves' diet. Rodents were captured from both the buffer zone and the core area using Sherman live traps [16,17] to determine the habitat quality for the EW. Six and eight square grids of 5625 m^2^ (75 m × 75 m) were established in the buffer zone and core area, respectively. A total of 2015 traps were set (834 traps in the buffer zone: 534 during the dry season and 300 during the wet season; 1181 traps in the core area: 431 during the dry season and 750 during the wet season). Trap stations were marked by coloured plastic tags on nearby vegetation to easily locate the traps during checking and collection. The traps were baited with peanut butter mixed with roasted barley flour and replenished each day. The traps were checked twice a day: during the early morning (6.30 to 8.30) and the late afternoon (16.30 to 18.30). They were set for a total of 75 h in each grid during both seasons. The abundance of each of the rodent species in both habitats were compared using the generalized linear model, a logit link and binomial distribution. We compared Shannon's diversity indices (*H*) of rodent and shrew species of the core area and buffer zone by fitting a generalized linear mixed-effects model using the lmer function in the lme4 package. Average Shannon's diversity for each one of the grids in 2015 trap sampling sessions from the two sites was used as the response variable, trapping locations as the random effect and habitat (buffer zone or core area) as the fixed effect. We estimated the abundance of the common mole rat, *Tachyoryctes splendens*, by counting active burrows in the buffer zone and the core area [13]. A total of 51 and 39 plots (20 m × 20 m) in the buffer zone and the core area, respectively, on the transects established for sighting AWs were randomly selected to be checked for active burrows. The mole rat abundances in the two study zones were compared using generalized linear mixed models with mole rat presence/abundance as response variables, plots as the random effect and habitat (buffer zone or core area) as the fixed effect.

## 3. Results

### 3.1. Wolf interactions

AWs intensively used the buffer zone (57.2%) and matrix (40.8%), but only rarely (2.0%) entered the core area (total sightings: *n* = 3052; electronic supplementary material, table S1). By contrast, EWs were observed mostly in the core area (82.4%), though occasionally in the buffer zone (18.6%) as well (total sightings: *n* = 252).

**Figure 3.**
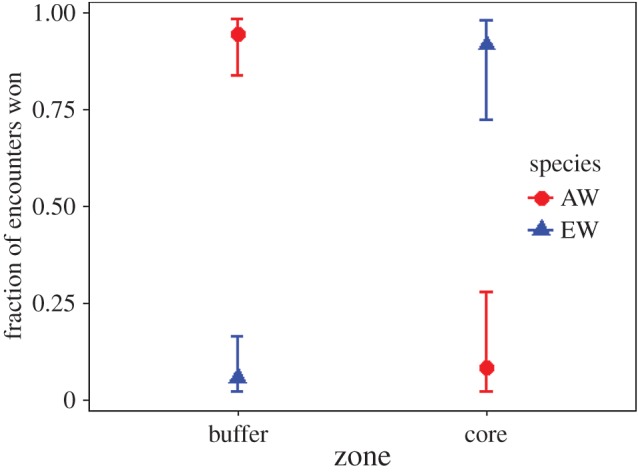
Fraction of agonistic encounters won by AWs and EWs in relation to encounter locations (buffer zone versus core area).

Within 12 months, we observed 82 interactions between AWs and EWs, of which 58 (70.7%) occurred in the buffer zone while 24 (29.3%) took place in the core area. With the exception of five neutral interactions (6.1%), all others were agonistic (93.9%). Of the 55 agonistic interactions in the buffer zone, 52 (94.5%) were won by AWs and only 3 (5.5%) by EWs (*Z* = −3.11, *p* = 0.002; figure 3). Conversely, of the 25 agonistic interactions in the core area, EWs won 23 (92.0%), whereas AWs won only 2 (8.0%) (*Z* = 5.42, *p* = 0.001). On three occasions (in the buffer zone), an AW not only chased but also bit an EW. The likelihood of AWs winning agonistic interactions increased with group size (*Z* = 2.45, *p* = 0.01), while group size had no effect on whether EWs won interactions (*Z* = 0.45, *p* = 0.12; table 1).

**Table 1.** Estimates of probability of the AW versus EW winning encounters in core area versus buffer zone. ‘Buffer zone’ was used as a reference level in the analysis.

effects	estimate	s.e.	*Z*	*p*
intercept	1.150	1.808	0.636	0.250
site (core versus buffer)	−8.971	3.043	−2.948	0.003
AW group size	3.171	1.295	2.449	0.014
EW group size	2.001	1.300	−1.590	0.124

The average duration per interaction was 3.1 ± 2.8 min (range 0.5–20 min; *n* = 82). The mean number of AWs involved per interaction was 1.9 ± 0.8 (range: 1–5), while EWs were more often solitary (mean 1.3 ± 0.4; range: 1–3).

### 3.2. Diet

Based on faecal analyses, rodents (47.5%; *n *= 642) were the top prey items of AWs, followed by livestock (cattle and sheep) remains obtained via predation or scavenging (17.2%), and by insects (11.2%), usually grasshoppers (table 2). Rodents occurred in 93.0% of EW scat samples [13], which is significantly higher than the proportion of rodents (47.2%) in AW scat samples (*t* = 4.939, *p* < 0.001).

**Table 2.** Seasonal differences in the frequency of occurrence (FO) of food items in the diet of African wolves at Guassa as determined by scat analysis.

	dry (*n* = 175)		wet (*n* = 175)		combined	
food items	*n*	% FO	*n*	% FO	*n*	% FO
rodents	164	53.2	137	42.0	301	47.2
Ethiopian hares (*Lepus fagani*)	5	1.6	9	2.8	14	2.2
livestock (hunted or scavenged)	24	7.8	85	26.1	109	17.2
duikers	0	0.0	3	0.9	3	0.5
wild birds	7	2.3	5	1.5	12	1.9
domestic chickens	0	0.0	7	2.1	7	1.1
unidentified bones	31	10.1	25	7.7	56	8.9
insects (mostly grasshoppers)	46	14.9	25	7.7	71	11.2
graminoids	14	4.5	3	0.9	17	2.7
potatoes	6	1.9	2	0.6	8	1.2
soil	11	3.5	25	7.7	36	5.7
total	308	100	326	100	634	100

### 3.3. Prey density

From trapping, we obtained 522 small mammals, including nine rodent and two shrew species (table 3). There were no significant differences in species abundance of small mammals except two species, *Lophuromys brevicaudus* and *Stenocephalemys albocaudata*, which have higher abundance in the core area (table 4). No significant difference in species diversity was found between the buffer zone and core areas (electronic supplementary material, table S2). The abundance of active burrows of mole rats, a primary prey item of EWs, did not vary significantly between the core area (mean = 0.57, s.d. = 0.50, *n* = 51) and the buffer zone (mean = 0.44, s.d. = 0.50, *n *= 39; *Z* = −1.24, *p* = 0.21; electronic supplementary material, table S3).

**Table 3.** Relative frequency (%) of rodent and shrew species trapped in the buffer zone and the core area during dry and wet seasons. Buffer zone: 834 traps, 534 dry season and 300 wet season; core area: 1181 traps: 431 dry season and 750 wet season.

	buffer zone		core area	
species	dry	wet	dry	wet
*Lophuromys brevicaudus*	3.56	9.33	12.06	20.80
*Stenocephalemys griseicauda*	5.81	0.00	0.00	2.13
*Stenocephalemys albipes*	4.87	0.00	0.00	0.00
*Stenocephalemys albocaudata*	0.00	7.33	3.25	7.73
*Mastomys natalensis*	2.43	0.00	0.00	0.00
*Otomys typus*	0.19	3.00	0.93	0.93
*Lophuromys flavopunctatus*	1.12	2.00	0.00	2.53
*Dendromus lovati*	0.00	0.00	0.23	0.00
*Arvicanthis abyssinicus*	0.37	0.00	0.00	0.00
*Crocidura baileyi*	0.00	0.00	0.23	2.40
*Crocidura macmillani*	0.37	0.00	0.23	1.33

**Table 4.** Rodent species abundance compared between the buffer zone and the core area (rodent species presence/absence as a response variable, habitat (buffer zone and core area) as fixed effect and traps as random variables). ‘Core area’ was used as a reference level in the analysis.

species		estimate	s.e.	*Z*	*p*
*Lophuromys brevicaudus*	intercept	−3.0699	0.1681	−18.257	<2 × 10^−16^
	core area	1.7036	0.183	9.307	<0.001
*Stenocephalemys griseicauda*	intercept	−4.2268	0.2908	−14.54	<2 × 10^−16^
	core area	0.393	0.3541	1.11	0.267
*Stenocephalemys albipes*	intercept	−3.6562	0.221	−16.543	<2 × 10^−16^
	core area	−0.3554	0.312	−1.139	0.255
*Stenocephalemys albocaudata*	intercept	−4.2268	0.2908	−14.536	<2 × 10^−16^
	core area	1.4923	0.3152	4.735	<0.001
*Mastomys natalensis*	intercept	−4.7719	0.3796	−12.572	<2 × 10^−16^
	core area	−0.3504	0.5364	−0.653	0.514
*Otomys typus*	intercept	−4.9273	0.4097	−12.027	<2 × 10^−16^
	core area	0.2604	0.5095	0.511	0.609
*Lophuromys flavopunctatus*	intercept	−4.9273	0.4097	−12.027	<2 × 10^−16^
	core area	0.8659	0.4677	1.852	0.0641
*Crocidura macmillani*	intercept	−6.725	1.001	−6.721	1.80 × 10^−11^
	core area	1.448	1.081	1.339	0.18

## 4. Discussion

Disease, including rabies and canine distemper virus, is the most immediate threat to the survival of EWs in Bale Mountains National Park, which contains the largest and most extensively studied population [18]. Our study at Guassa reveals interspecific competition as another potential threat to EWs.

AWs inhabit the buffer zone and surrounding human-dominated landscape at Guassa, while EWs predominantly inhabit the core area of the protected area. AWs dominated interactions in the buffer zone, whereas EWs dominated in the core area, indicating that both species defended their territories. The outcome of interactions was affected by territorial dominance and numerical superiority which played a more important role than body size differences. Group size advantage in interspecific competition is common in carnivores [19–21]. Although no intraguild predation or prolonged physical fighting was observed, the three occasions of brief physical contact we observed involved biting, revealing the potential for interspecific disease transmission. Close spatial proximity also increases the likelihood of hybridization [22,23].

The EW is a rodent specialist which mainly foraged in the core area, while the AW is an opportunistic forager that regularly consumes livestock, and is thus relatively tolerant of habitat alteration by humans. Nevertheless, although AWs sometimes preyed on livestock and fed on insects and other non-rodent foods, rodents were still the top food item of AWs. This finding suggests the possibility of exploitative competition between AWs and EWs. Rodent abundances and species compositions did not differ significantly between the core area and the buffer zone, suggesting that, in the absence of AWs, EWs could also exploit the buffer zone habitat, which could facilitate an increase in EW population size. This result implies that the use of degraded habitats by EWs may be constrained by interspecific competition, and not merely by the absence of suitable habitat, and thus prevent EWs from range expansion and population growth.

## 5. Conclusion

AWs predominantly inhabit the buffer zone, a human-dominated landscape, while EWs mostly use the more intact core area at Guassa. The diets of EWs and AWs overlap, but AWs exhibit much wider dietary breadth. Patterns of interspecific interaction imply that each species engages in territorial defence against the other. Our study calls attention to the behavioural mechanisms that underlie competition between EWs and AWs, suggesting that increasing human encroachment and habitat loss may offer AWs a competitive advantage over EWs. EW conservation efforts would thus benefit from concurrent monitoring of AW populations where the two taxa co-occur.

## Supplementary Material

Tables S1 - S3

## Data accessibility

Data available at the Dryad Digital Repository: (http://dx.doi.org/10.5061/dryad.vj2nn) [24].
