# Supplementary material for: Competition between sympatric wolf taxa: an example involving African and Ethiopian wolves
Source: R Soc Open Sci. 2018 May 2;5(5):172207. doi: 10.1098/rsos.172207 (PMC5990763; doi:10.1098/rsos.172207)
Supplement: Tables S1 - S3 [file rsos172207supp1.docx]

Table S1. Impact of the number of individuals of each wolf species present on the outcome of interactions.

| Number of  wolves  EW> AW | Winner (number of wins by each species) | | | |
| --- | --- | --- | --- | --- |
|  | EW | AW | Neutral | Total |
|  |  |  |  |  |
| Buffer | 1 | 1 | 0 | 2 |
| Core | 4 | 0 | 0 | 4 |
| AW>EW |  |  |  |  |
| Buffer | 0 | 29 | 0 | 29 |
| Core | 6 | 2 | 0 | 8 |
| AW=EW |  |  |  |  |
| Buffer | 2 | 20 | 5 | 27 |
| Core | 12 | 0 |  | 12 |
| Total | 25 | 52 | 5 | 82 |

Table S2. Estimates, standard errors (SE) and P-values from a linear mixed effects model with Shannon diversity index (H) as the response variable, Grids as the random effect and habitat (buffer zone or core area) as the fixed effect.

|  | Estimate | SE | df | t | P |
| --- | --- | --- | --- | --- | --- |
| Intercept | 0.0495 | 0.0207 | 12.17 | 2.39 | 0.034 |
| core area | 0.0200 | 0.0273 | 12.08 | 0.73 | 0.478 |

Table S3. Estimates of mole rat presence in the buffer zone and core Zone of Guassa Mountains from generalized linear model with mole rat presence/abundance as response variables, plots as the random effect and habitat (buffer zone or core zone) as the fixed effect.

|  | Estimate | SE | Z | P |
| --- | --- | --- | --- | --- |
| (Intercept) | 1.14e-15 | 3.02e-01 | 0.00 | 1.00 |
| Mole rat pressene | -5.34e-01 | 4.29e-01 | -1.24 | 0.21 |
